# SUMO Modification Stabilizes Dengue Virus Nonstructural Protein 5 To Support Virus Replication

**DOI:** 10.1128/JVI.00223-16

**Published:** 2016-04-14

**Authors:** Chan-I Su, Chung-Hsin Tseng, Chia-Yi Yu, Michael M. C. Lai

**Affiliations:** a Department of Microbiology and Immunology, College of Medicine, National Cheng Kung University, Tainan, Taiwan; b Center of Infectious Disease and Signaling Research, National Cheng Kung University, Tainan, Taiwan; c Department of Medical Laboratory Science and Biotechnology, College of Medicine, National Cheng Kung University, Tainan, Taiwan; d Institute of Molecular Biology, Academia Sinica, Taipei, Taiwan; e Research Center for Emerging Viruses, China Medical University Hospital, Taichung, Taiwan; University of Southern California, Keck School of Medicine

## Abstract

Small ubiquitin-like modifier (SUMO) participates in a reversible posttranslational modification process (SUMOylation) that regulates a wide variety of cellular processes and plays important roles for numerous viruses during infection. However, the roles of viral protein SUMOylation in dengue virus (DENV) infection have not been elucidated. In this study, we found that the SUMOylation pathway was involved in the DENV life cycle, since DENV replication was reduced by silencing the cellular gene *Ubc9*, which encodes the sole E2-conjugating enzyme required for SUMOylation. By *in vivo* and *in vitro* SUMOylation assays, the DENV NS5 protein was identified as an authentic SUMO-targeted protein. By expressing various NS5 mutants, we found that the SUMO acceptor sites are located in the N-terminal domain of NS5 and that a putative SUMO-interacting motif (SIM) of this domain is crucial for its SUMOylation. A DENV replicon harboring the SUMOylation-defective SIM mutant showed a severe defect in viral RNA replication, supporting the notion that NS5 SUMOylation is required for DENV replication. SUMOylation-defective mutants also failed to suppress the induction of STAT2-mediated host antiviral interferon signaling. Furthermore, the SUMOylation of NS5 significantly increased the stability of NS5 protein, which could account for most of the biological functions of SUMOylated NS5. Collectively, these findings suggest that the SUMOylation of DENV NS5 is one of the mechanisms regulating DENV replication.

**IMPORTANCE** SUMOylation is a common posttranslational modification that regulates cellular protein functions but has not been reported in the proteins of dengue virus. Here, we found that the replicase of DENV, nonstructural protein 5 (NS5), can be SUMOylated. It is well known that providing RNA-dependent RNA polymerase activity and antagonizing host antiviral IFN signaling are a “double indemnity” of NS5 to support DENV replication. Without SUMOylation, NS5 fails to maintain its protein stability, which consequently disrupts its function in viral RNA replication and innate immunity antagonism. DENV threatens billions of people worldwide, but no licensed vaccine or specific therapeutics are currently available. Thus, our findings suggest that rather than specifically targeting NS5 enzyme activity, NS5 protein stability is a novel drug target on the growing list of anti-DENV strategies.

## INTRODUCTION

Dengue virus (DENV) is an emerging mosquito-borne virus (1) that causes a variety of clinical symptoms in human beings, ranging from self-limited febrile dengue fever to life-threatening dengue hemorrhagic fever and dengue shock syndrome (2). Conventionally, there are at least four distinct DENV serotypes (DENV1 to DENV4). The interactions between the host and these closely related viruses further complicate this infectious disease. Despite the fact that DENV infection poses an increasing threat to worldwide public health, neither vaccines nor antiviral drugs are available (3). DENV is a single-stranded, positive-sense RNA virus of the family Flaviviridae. The DENV genome encodes a single polyprotein precursor, which is processed into three structural proteins (C, prM, and E) and seven nonstructural proteins (NS1, NS2A, NS2B, NS3, NS4A, NS4B, and NS5). Among them, the NS5 protein is the viral RNA-dependent RNA polymerase (RdRp) (4) and also subverts STAT2-mediated antiviral interferon (IFN) signaling of host innate immunity (5, 6).

NS5 is the largest (∼100 kDa) and most conserved protein among DENV viral proteins. In addition to RdRp enzymatic activities, it also contains methyltransferase (MTase) (4, 7–9). Furthermore, a nuclear localization signal (NLS) domain, which is responsible for the nuclear transport of DENV NS5, has been found between the MTase and RdRp domains despite the fact that DENV replicates in the cytoplasm (10). Thus, DENV NS5 is predominantly localized in the nucleus (11, 12). DENV2 NS5 protein has been shown to be modified posttranslationally by phosphorylation; different levels of phosphorylation within at least 4 distinct serine residues would decide the subcellular distribution of NS5 protein in infected cells (13).

The small ubiquitin-like modifier (SUMO) protein is a molecule of 11 kDa that covalently binds to lysine residues of target protein (SUMOylation) (14) in a reversible manner (15, 16). Among the four SUMO isoforms (SUMO1 to SUMO4) identified in mammals, SUMO2 and SUMO3 share 97% sequence identity and often are referred to as SUMO2/3. In contrast to SUMO2/3, SUMO1 is different in structure and charge distribution, which reflects the low (50%) identity shared between SUMO1 and SUMO2/3 (17). This might explain the distinct subcellular location of SUMO1 from that of SUMO2/3: SUMO1 is located in the nuclear envelope, nucleolus, and cytoplasmic foci, whereas SUMO2/3 accumulates on chromosomes at an earlier point in nuclear reformation processes (18). However, the role of SUMO4 is still an enigma (19). As a conserved member of protein modifications, two *SUMO* paralogs, *SUMO1* and *SUMO3*, have been predicted in insects (20), but the whole picture of SUMOylation in insects remains to be elucidated.

The functional consequences of protein SUMOylation might result from inducing a conformational change of the targeting protein, hindering or creating binding sites to its interactors (21). Depending on the target proteins, SUMO modification regulates a wide variety of cellular processes, including protein-protein interaction, protein-DNA interaction, altering protein localization, and the stabilizing of modified proteins (22–25). SUMOylation is carried out by an E1-activating enzyme, an E2-conjugating enzyme (Ubc9), and one of several SUMO E3 ligases (26, 27). Approximately 75% of SUMO target proteins are modified within a consensus motif consisting of ψKxD/E (where ψ is a large hydrophobic residue, K is the modified lysine, and x is any residue) (19), but SUMO could modify target proteins beyond this motif as well. A hydrophobic core of V/I-X-V/I-V/I, flanked by a cluster of negatively charged residues, was characterized as the SUMO-interacting motif (SIM) that binds to the interacting surface of SUMO. Therefore, proteins containing SIMs are expected to be the key regulators governing SUMOylation processes and biological functions of SUMOylated proteins (21).

In contrast to the sophisticated understanding of cellular protein SUMOylations, how SUMOylation is regulated and how it regulates virus replication during infection has just started to be delineated. Recently, the posttranslational modification of viral proteins was recognized as an important regulatory mechanism for various viral functions (28, 29). Studies on influenza virus (IAV) and hepatitis delta virus (HDV) also showed the importance of SUMO modifications of viral proteins in viral replication (30, 31). Specifically, SUMOylation of the small form of hepatitis delta antigen protein selectively enhanced HDV genomic RNA synthesis (30). The conjugation of SUMO to IAV matrix protein M1 is required for the interaction between M1 and viral RNP; thus, the failure in M1 SUMOylation prevents maturation and assembly of IAV (31). Also, DENV envelope protein E has been reported to interact with SUMO-conjugating enzyme Ubc9 in an overexpression system. However, SUMOylation of this protein had not been verified experimentally (32). In fact, none of the DENV proteins had been proven to be the SUMO-targeting protein, and the exact role of SUMOylation in DENV infection remains unresolved. In this study, we showed that the SUMO modification pathway is involved in DENV replication in host cells (32–34). We demonstrated that DENV NS5, the replicase responsible for viral genome replication, is a SUMO target protein. A putative SIM motif was found in the N-terminal domain of NS5 and is required for NS5 SUMOylation. Importantly, the SIM-mutated NS5 that failed to be modified by SUMO showed attenuated abilities in both supporting viral RNA replication and antagonizing IFN signaling. We also found that the degradation of SIM-mutated NS5 was faster than that of the wild-type protein. Collectively, these data indicate that SUMOylation fine-tunes NS5 protein stability to regulate its functions, such as viral RNA replication and the antagonism of host innate immunity.

## MATERIALS AND METHODS

### Cell culture and transfection.

The human embryonic kidney 293T (HEK293T) and human hepatoma Huh7 cell lines were cultured in Dulbecco's modified Eagle's medium (DMEM) supplemented with 10% fetal bovine serum (FBS). A549, a human lung carcinoma cell line, was cultured in F12K medium supplemented with 10% FBS. Mouse neuroblastoma cell line N18 (35) was cultured in RPMI with 5% FBS. All cell lines were incubated at 37°C in 5% CO_2_. BHK-21 cells harboring DENV2 replicon (36) were cultured in DMEM with 10% FBS and selected by 500 μg/ml G418. Plasmid DNA and DENV RNA transfection experiments were performed by PolyJet (SignaGen Laboratories) and DMRIE-C (Invitrogen), respectively, according to the manufacturer's instructions. The Huh7 cells stably expressing control short hairpin RNA (shRNA) (Ctrl) or shRNA targeting Ubc9 have been described elsewhere (31).

### siRNA and shRNA depletion.

Small interfering RNAs (siRNAs) targeting human Ubc9 (siUBE2I; no. L-004910-00-0005), DENV serotype 2 (siDENV2; no. L-007189-00-005), and nontargeting pool (siCtrl; no. D-001810-10-05) were purchased from Dharmacon. As a smart pool, siUBE2I contains the four oligonucleotides 5′-GGGAAGGAGGCUUGUUUAA-3′, 5′-GAAGUUUGCGCCCUCAUAA-3′, 5′-GGCCAGCCAUCACAAUCAA-3′, and 5′-GAACAACCAUUAUUUCACC-3′, whereas the siDENV2 pool includes 5′-CAGGAAAGACGAAAAGAUAUU-3′ and 5′-UAUCUUUUCGUCUUUCCUGUU-3′. siRNAs were delivered by Lipofectamine 2000 (Invitrogen) according to the manufacturer's instructions. Lentiviral vectors with shRNAs targeting human UBE2I (5′-GCCTACACGATTTACTGCCAA-3′; TRCN0000007205) or LacZ (5′-CGCGATCGTAATCACCCGAGT-3′; TRCN0000072224) were from the National RNAi Core Facility, Taiwan. The preparation of lentivirus followed the protocol from the National RNAi Core Facility (Academia Sinica, Taiwan).

### Plasmids.

All 10 of the DENV2 viral proteins derived from strain PL046 were subcloned into a pcDNA3.1-2HA vector (encoding hemagglutinin [HA] tag at both ends of the multiple cloning site). Plasmids expressing NS5 mutants, including lysine-to-arginine (K-R) and deletion mutants, were derived from pcDNA3.1-HA-NS5-HA by PCR-based site-directed mutagenesis or jumping PCR methods. The introduction of single or multiple K-R substitution mutations in full-length NS5 was performed by jumping PCR with the primers 5′AgeI (5′-GAGAAATGGAAAAACCGGTTGAAC-3′) and 3′PmlI (5′-TTCAACAGCCTCACGTGCCGACTT-3′) plus the K-R substitution primers. The verified K-R substitution sequence was cloned back into pcDNA3.1-HA-NS5-HA by using AgeI and PmlI restriction enzymes. Primers for deletions and mutations of NS5 are listed in Table 1. pDENV-Luc replicon was kindly provided by Huey-Nan Wu (Institute of Molecular Biology, Academia Sinica) (36). The expression plasmids pcDNA3.1-V5-Ubc9, pEGFP-SUMO, pcDNA3-HA-SENP1, and pcDNA3-HA-SENP2 were kind gifts from Hsiu-Ming Shih (Institute of Biomedical Sciences, Academia Sinica).

**TABLE 1:** Primers used in DENV protein cloning or mutagenesis

Purpose	Name	Sequence
Individual DENV2 protein expression		
Core	F-EcoRI	5′-GGAATTCATGATGAATAACCAACGGAAAAAGGCG-3′
	R-NotI	5′-AAGGAAAAAAGCGGCCGCCCGCCATCACTGTTGGAATCAGCAT-3′
NS1	F-NotI	5′-AAGGAAAAAAGCGGCCGCATGGATAGTGGTTGCGTTGTGAGCTGG-3′
	R-KpnI	5′-GGGGTACCCGGCTGTGACCAAGGAGTTGACCAA-3′
NS2A	F-EcoRI	5′-GGAATTCATGGGACATGGGCAGATTGACAACTTC-3′
	R-NotI	5′-AAGGAAAAAAGCGGCCGCCCCTTTTCTTGCTAGTTCGTGAAAG-3′
NS2B	F-EcoRI	5′-GGAATTCATGAGCTGGCCACTAAATGAGGCTATC-3′
	R-NotI	5′-AAGGAAAAAAGCGGCCGCCCCGTTGTTTCTTCACTTCCCACAG-3′
NS3	F-NotI	5′-AAGGAAAAAAGCGGCCGCATGGCTGGAGTATTGTGGGATGTCCCT-3′
	R-KpnI	5′-GGGGTACCCACTTTCTTCCAGCTGCAAATTCCTT-3′
NS4A	F-EcoRI	5′-GGAATTCATGTCCTTGACCCTGAACCTAATCACA-3′
	R-NotI	5′-AAGGAAAAAAGCGGCCGCCTCTTTTCTGAGCTTCTCTAGTTGC-3′
NS4B	F-EcoRI	5′-GGAATTCATGGCAGCAGCGGGCATCATGAAAAAC-3′
	R-NotI	5′-AAGGAAAAAAGCGGCCGCCCCTTCTTGTATTGGTTGTGTTCTT-3′
NS5	F-NotI	5′-AAGGAAAAAAGCGGCCGCATGGGAACTGGCAACATAGGAGAGACG-3′
	R-NotI	5′-AAGGAAAAAAGCGGCCGCCCCACAGGACTCCTGCCTCTTCCTC-3′
DENV2 NS5 truncation mutants		
N1	F-1∼627	5′-AAGGAAAAAAGCGGCCGCATGGGAACTGGCAACATAGGAGAGACG-3′
	R-1∼627	5′-AAGGAAAAAAGCGGCCGCCTCCTTCTCCTTCCATCTGTCT-3′
N2	F-1∼450	5′-AAGGAAAAAAGCGGCCGCATGGGAACTGGCAACATAGGAGAGACG-3′
	R-1∼450	5′-ACCCGGGACTCGAGGCGGCCGCACATGTTTCACACTTTCCTTC-3′
C1	F-71∼900	5′-AAGGAAAAAAGCGGCCGCATGGTCACACCAGAAGGGAAAGTA-3′
	R-71∼900	5′-AAGGAAAAAAGCGGCCGCCCCACAGGACTCCTGCCTCTTCCTC-3′
C2	F-301∼900	5′-AAGGAAAAAAGCGGCCGCATGACATGGGCTTACCATGGTAGC-3′
	R-301∼900	5′-AAGGAAAAAAGCGGCCGCCCCACAGGACTCCTGCCTCTTCCTC-3′
C3	F-301∼705	5′-AAGGAAAAAAGCGGCCGCATGACATGGGCTTACCATGGTAGC-3′
	R-301∼705	5′-AAGGAAAAAAGCGGCCGCCTTGTGTCCAGTCGCTCCACCC-3′
C4	F-301∼545	5′-AAGGAAAAAAGCGGCCGCATGACATGGGCTTACCATGGTAGC-3′
	R-301∼545	5′-AAGGAAAAAAGCGGCCGCCTTCCAGTGTGATTCTTGTGTC-3′
DENV2 NS5 K-R substitution		
K139R	F	5′-TTCTTTACTCCGCCAGAAAGGTGCGACACATTGCT-3′
	R	5′-AGCAATGTGTCGCACCTTTCTGGCGGAGTAAAGAA-3′
K181R	F	5′-TGCATAAGGGTTCTCAACCCATACATGCCC-3′
	R	5′-GAGAACCCTTATGCAAAATTGGATGTTGTTGTT-3′
K193R	F	5′-CCATACATGCCCTCAGTCATAGAAAGAATGGAAGCACTAC-3′
	R	5′-GTAGTGCTTCCATTCTTTCTATGACTGAGGGCATGTATGG-3′
K200R	F	5′-GGAAGCACTACAAAGGAGATATGGAGGAGCCTTAG-3′
	R	5′-CTAAGGCTCCTCCATATCTCCTTTGTAGTGCTTCC-3′
K285R	F	5′-GAAAAAATAAGACAAGAGCATGAAACATCATG-3′
	R	5′-TCTTATTTTTTCTATTCTTTTCCCAATTATGTCCAG-3′
K101/105R	F	5′-GAAGTCAGAGGCCTGACAAGAGGA-3′
	R	5′-CAGGCCTCTGACTTCTCTTACATT-3′
K246/248/249R	F	5′-ATGAGACACAGGAGAGCCACTTACGAGCCA-3′
	R	5′-GGCTCTCCTGTGTCTCATTGTGAATCTATTG-3′
K279/283/285R	F	5′-GGGAGAAGAATAGAAAGAATAAGACAAGAGCAT-3′
	R	5′-TCTTATTCTTTCTATTCTTCTCCCAATTATGTCCAG-3′
SIMmut	F	5′-AAAGCAGCGGCCGCTGGTTGCGGCAGAGGA-3′
	R	5′-ACCAGCGGCCGCTGCTTTCCCTTCTGGTGT-3′

### *In vivo* and *in vitro* SUMOylation assays.

For the *in vivo* SUMOylation assay, plasmids expressing HA-tagged DENV proteins and V5-tagged Ubc9 were transiently cotransfected into HEK293T cells with or without the plasmid expressing enhanced green fluorescent protein (EGFP)-tagged SUMO1. After 48 h of transfection, the transfectants were lysed by radioimmunoprecipitation assay (RIPA) buffer (150Mm NaCl, 1% NP-40, 0.5% sodium deoxychloride, 0.1% SDS, 50 mM Tris, pH 7.5) containing 20 mM *N*-ethylmaleimide (NEM) and subjected to immunoprecipitation by anti-HA agarose (Sigma). Viral proteins and their corresponding SUMOylated species were analyzed by performing immunoblotting with SDS-PAGE.

For the *in vitro* SUMOylation assay, a SUMOylation kit (Biomol) was used according to the manufacturer's instructions. HA-tagged NS5 proteins were purified from HEK293T cells transfected with pcDNA3.1-HA-NS5-HA by anti-HA agarose. Five microliters of HA-tagged NS5 proteins was mixed with 2 μl of 10× SUMO buffer, 1 μl of 20× SUMO activating enzyme solution (SUMO E1), and 1 μl of 20× SUMO conjugating enzyme solution (SUMO E2), with or without 1 μl of 20× Mg-ATP solution. Water was added to a final volume of 20 μl. The reactions were conducted at 30°C for an hour, stopped by boiling at 95°C for 5 min with SDS-PAGE loading buffer, and analyzed by Western blotting.

### Reporter assay.

A549 cells were cotransfected with the viperin promoter-driven reporter Vip-Luc (37) (0.3 μg), pRL-TK (0.1 μg), and WT or SIM-mutated DENV NS5 expression plasmids (0.5 μg). At 16 h posttransfection, cells were treated with IFN-α2a (500 U; Roferon-A; Roche) for another 8 h. The cells were harvested and analyzed by a dual-luciferase assay system (Promega).

### *In vitro* transcription.

pDENV-Luc-replicon plasmids harboring WT or SIM-mutated NS5 were linearized by the restriction enzyme PmeI and were purified by phenol-chloroform. The *in vitro* transcription assay was carried out with a T7 mMESSAGE mMACHINE Ultra (Ambion). Linearized replicon templates were mixed with 2× NTP/CAP, 10× T7 buffer, T7 enzyme, and 30 mM GTP. After 4 h of incubation at 37°C, DNase was added to the reaction mixture for another 30 min. The synthetic RNA finally was cleaned up by a MEGAclear RNA purification kit (Ambion).

### Western blot analysis.

Cells were lysed with RIPA buffer containing a cocktail of protease (Sigma). Protein samples were separated by SDS-PAGE and transferred to a polyvinylidene difluoride (PVDF) transfer membrane (Hybond-P; GE Healthcare). The nonspecific antibody binding sites were blocked with milk in Tris-buffered saline–Tween 20 and then reacted with the primary antibodies anti-HA (GTX29110 [GeneTex] or 3724 [Cell Signaling]), anti-Ubc9 (4786; Cell Signaling), anti-actin (A5441; Sigma), anti-GFP (GTX26556GeneTex), anti-STAT1 (9175; Cell Signaling), anti-STAT2 (05-693; Upstate), and anti-RIG-I (3743; Cell Signaling). Anti-NS5 and -NS3 antibodies are gifts from Huey-Nan Wu. Blots were treated with horseradish peroxidase-conjugated secondary antibody (Thermo Scientific), and signals were detected by enhanced chemiluminescence (ECL; Millipore).

## RESULTS

### Silencing of the SUMO E2 enzyme Ubc9 represses DENV replication.

**FIG 1:**
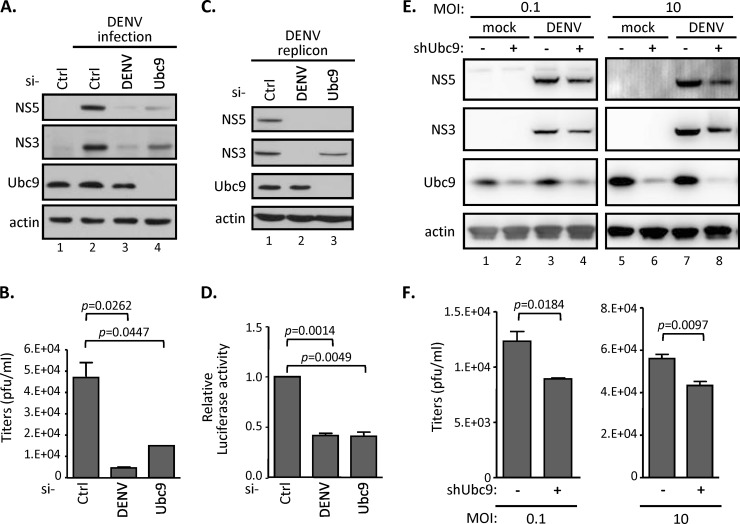
Silencing SUMO E2 enzyme Ubc9 represses DENV replication. (A and B) A549 cells were transfected with the indicated siRNAs for 48 h and then infected with DENV serotype 2 (MOI of 0.1) for another 48 h. Intracellular protein expression levels and infectious DENV titer in the culture supernatant were measured by Western blotting (WB) (A) and by plaque forming assay (B), respectively. Data are expressed as means and standard deviations (SD) (*n* = 3 per group) and were compared to those for siCtrl by two-tailed Student's *t* test. (C and D) A stable BHK-21 cell line harboring DENV2 replicon were transfected with the indicated siRNAs for 48 h. WB (C) and luciferase assay (D) were carried out to analyze the indicated protein expression levels and the replication levels of DENV2 replicon. (E and F) A549 cells stably expressing shRNA targeting control *LacZ* or *Ubc9* were infected with DENV (MOI, 0.1 and 10) for 24 h. Cell lysates were analyzed by WB with the indicated antibodies (E), and culture supernatants were harvested for virus titration by performing plaque assays (F). The error bars represent the means and SD (*n* = 3 per group) and were compared by Student's *t* test.

Recent evidence indicated that the cellular SUMO conjugase Ubc9, a key component for protein SUMOylation, interacts with several dengue viral proteins (32, 34, 38). To determine whether the SUMO modification pathway is involved in DENV replication, we suppressed the SUMOylation system by knocking down Ubc9 expression through RNA interference (RNAi) silencing approaches. In A549 cells transfected with Ubc9-specific siRNA, the expression of Ubc9 was substantially suppressed; correspondingly, the production of viral nonstructural proteins (represented by NS3 and NS5) also was substantially decreased after DENV infection (Fig. 1A). For comparison, the transfection of the DENV-specific siRNA into DENV-infected cells showed an almost complete loss of viral replication. Consistent with this, culture media derived from DENV-infected siUbc9 cells also showed a lower viral titer than that from the control siRNA-transfected cells (Fig. 1B). To further understand the mechanism of the involvement of the cellular SUMO modification system in the DENV replication processes, we used a stable cell line, BHK21-DENV2-SGR, containing a replication-competent DENV subgenomic RNA replicon, which expresses viral nonstructural proteins and core protein. A firefly luciferase reporter gene was fused in frame with the DENV replicon to monitor the viral RNA translation and replication statuses (36). We found that both viral protein expression (Fig. 1C) and replicon luciferase activity (Fig. 1D) were inhibited by silencing endogenous Ubc9 expression in BHK21-DENV2-SGR cells. The human A549 cells harboring shRNA that stably knocked down endogenous Ubc9 displayed similar characteristics upon DENV infection. We found that both the expression of viral proteins (Fig. 1E) and the production of viral progeny (Fig. 1F) were slightly reduced in shUbc9 cells upon DENV infection at either a low or high multiplicity of infection (MOI). Despite the incomplete knockdown of Ubc9 in A549 cells (Fig. 1E), these results consistently indicated that the cellular SUMO modification system is involved in the DENV life cycle.

### DENV NS5 protein is a target for SUMOylation.

**FIG 2:**
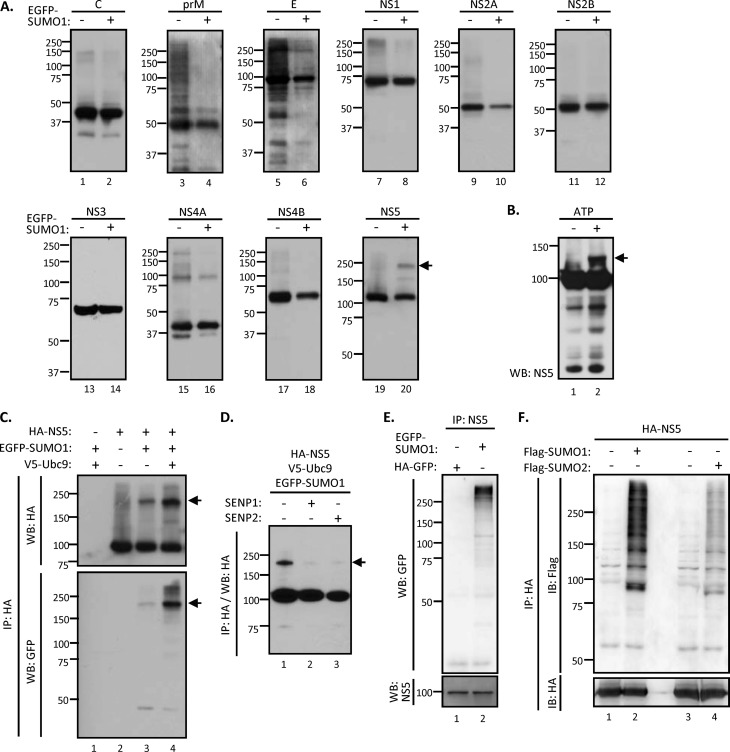
DENV NS5 protein is a target for SUMO modification. (A) HEK293T cells were cotransfected with Ubc9 and each HA-tagged DENV viral protein, with or without EGFP-tagged SUMO1, for 48 h. The cell lysates were subjected to immunoprecipitation (IP) by anti-HA agarose and WB analysis with anti-HA antibody. (B) The *in vitro* SUMOylation assay was reconstituted with recombinant SUMO E1 (Aos1/Uba2), SUMO E2 (Ubc9), His-tagged SUMO1, and immunopurified NS5 in the presence (+) or absence (−) of ATP as described in Materials and Methods. The reaction mixture was analyzed by WB with anti-NS5 antibody. (C) HEK293T cells were cotransfected with NS5, SUMO1, and Ubc9 or left untransfected, as indicated, for 48 h. Transfectants were collected and subjected to analysis by IP-WB with the indicated antibodies. (D) HEK293T cells were cotransfected with NS5, SUMO1, and Ubc9 together with SENP1 or SENP2 for 48 h as indicated. Cell lysates were harvested for IP-WB analysis with anti-HA antibody. Arrow, SUMOylated NS5 protein. (E) N18 cells were infected with DENV (MOI of 10) for 6 h and then transfected with EGFP-tagged SUMO1 or HA-tagged GFP. After 18 h of transfection, cells were lysed and analyzed by IP-WB with anti-GFP and anti-NS5 antibodies. (F) HEK293T cells were cotransfected with NS5 and Ubc9 plus Flag-tagged SUMO1 or SUMO2 for 48 h. Transfectants were harvested and subjected to analysis by IP-WB with the indicated antibodies. IB, immunoblot.

Since Ubc9 previously has been shown to interact with several DENV proteins (32, 34, 38), we asked whether any viral protein could be modified by SUMO. To this end, all 10 of the DENV proteins each were cloned with an HA tag and used in an *in vivo* SUMOylation assay to screen the potential SUMOylated DENV proteins. By cotransfection with EGFP-tagged SUMO1, each HA-tagged viral protein was immunoprecipitated by anti-HA agarose and separated by SDS-PAGE to reveal any additional protein bands showing a higher molecular mass than those expected of each viral protein. Among all of the viral proteins we examined, a higher-molecular-mass band was found only with NS5 (Fig. 2A), which suggested that DENV NS5 can be SUMOylated. E and NS4A also showed several higher-molecular-mass bands, both in the absence and in the presence of SUMO. The nature of these proteins is not known. For the current study, we focused on NS5.

We performed an *in vitro* SUMOylation assay to evaluate whether DENV NS5 is a direct target for SUMO1 modification. In a minimal SUMOylation reaction reconstitution mixture containing immunopurified NS5 protein, SUMO-activating enzyme mixture (Aos1/Uba2; E1), Ubc9 (E2), and His-tagged SUMO1 (His-SUMO1), a higher-molecular-mass species migrated slower than the bulk of NS5 protein (Fig. 2B). This band was detected only in the presence of ATP and may represent a SUMOylated NS5. This result indicates that NS5 can be SUMOylated *in vitro* in an ATP-dependent manner.

To ascertain that Ubc9 is responsible for the SUMOylation of NS5 in mammalian cells, an *in vivo* SUMOylation assay was performed. HA-tagged NS5 and the various components of SUMOylation machinery, including EGFP-tagged SUMO1, were cotransfected into HEK293 cells. A higher-molecular-mass species was detected by immunoblotting against either HA or GFP, suggesting that NS5 was SUMOylated (Fig. 2C, lane 3). Its amount was increased by the overexpression of Ubc9 (Fig. 2C, lane 4). The molecular size of this protein is estimated to be approximately 180 kDa. Since the predicted sizes of HA-tagged NS5 protein and EGFP-tagged SUMO1 are 103 kDa and 40 kDa, respectively, these results suggested that NS5 is modified by two SUMO molecules (Fig. 2C).

SUMOylation is a highly dynamic process that can be reversed by the action of SUMO proteases. Of the six human SUMO proteases (SENP1-3 and SENP5-7), SENP1 and SENP2 are responsible for the deSUMOylation of SUMO1-conjugated proteins (39, 40). To further confirm that NS5 is SUMOylated, SENP1 or SENP2 was added to the *in vivo* SUMOylation assay. The result showed that the band intensity of the higher-molecular-mass NS5 species was drastically reduced by the presence of either SENP1 or SENP2 (Fig. 2D). This result established that the 180-kDa band represents the SUMOylated NS5. To further understand whether NS5 can be modified by SUMOylation upon DENV infection, N18 cells were transfected with EGFP-tagged SUMO1 or control HA-GFP for 6 h, followed by DENV infection. We found that EGFP-SUMO1 but not HA-GFP was readily conjugated to DENV NS5 by immunoprecipitation using anti-NS5 antibody (Fig. 2E). To understand whether DENV NS5 can be modified by other SUMO species, an *in vivo* SUMOylation assay was performed by using Flag-tagged SUMO1 or SUMO2. We found that Flag-SUMO1 readily conjugated to NS5, whereas Flag-SUMO2 showed a much lower conjugation efficiency (Fig. 2F). Taken together, these data supported the notion that NS5 is an authentic target for SUMOylation, and we will focus on the relationship between NS5 and SUMO1 for the remainder of this study.

### The putative SUMO-interacting motif located in the N terminus of NS5 is crucial for NS5 protein SUMOylation.

**FIG 3:**
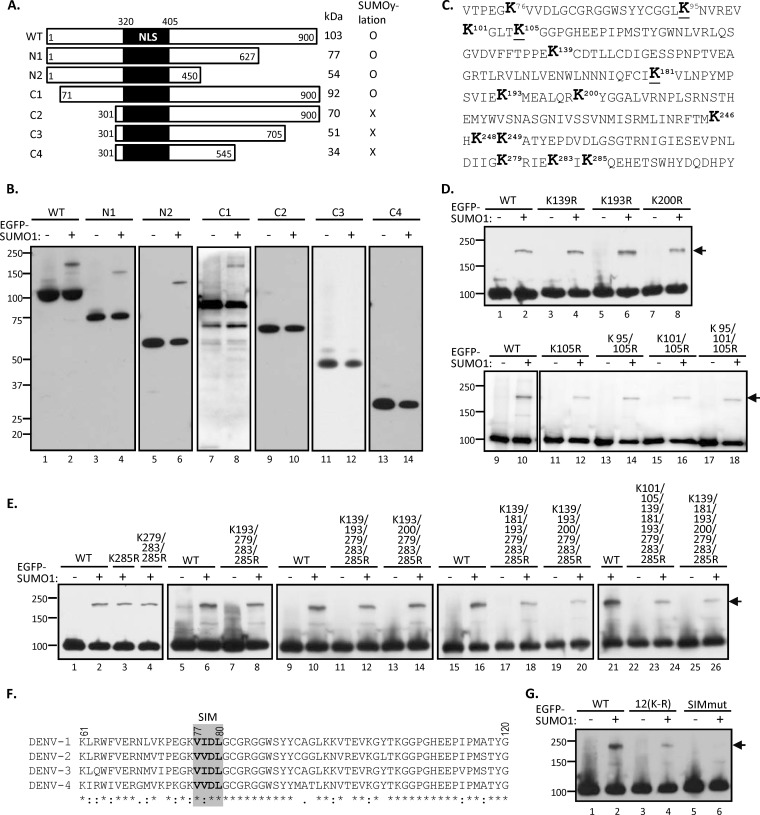
Putative SUMO-interacting motif (SIM) located in the N-terminal domain of NS5 is crucial for NS5 SUMOylation. (A) Schematic diagram of NS5 and its truncation derivatives. The predicted sizes and the ability of NS5 derivatives to undergo SUMOylation are summarized on the right. NLS, nuclear localization signal. (B) HEK293T cells were cotransfected with Ubc9 and each NS5 construct with (+) or without (−) EGFP-tagged SUMO1 for 48 h. Transfectants were harvested and analyzed by IP-WB using anti-HA antibody. (C) Amino acid sequence of the N-terminal 71 to 300 residues in DENV2 NS5. The lysine (K) residues are shown in boldface, whereas the conserved K residues among four serotypes of DENV are underlined. (D) In HEK293T cells, plasmid expressing NS5-WT or the indicated mutants containing single, double, or triple K-R substitution(s) were cotransfected with or without EGFP-SUMO1 and Ubc9. The whole-cell extracts then were collected and subjected to IP-WB analysis by anti-HA antibody. (E) HEK293T cells were cotransfected with Ubc9, EGFP-SUMO1, and each NS5 construct containing multiple K-R substitutions as indicated for 48 h. Transfectants were harvested and analyzed by IP-WB using anti-HA antibody. (F) Protein alignment of the N-terminal sequences from four different serotypes of DENV NS5. The putative SIM of DENV NS5 is shown in the gray square. (G) HEK293T cells were cotransfected with Ubc9 and/or EGFP-SUMO1 plus NS5-WT or its mutant containing 12 K-R substitutions [termed 12(K-R)] or VVDL-to-AAAA substitution at the putative SIM motif (SIMmut). Cells were harvested at 48 h posttransfection for IP-WB analysis using anti-HA antibody. Arrow, SUMOylated NS5 proteins.

SUMOpylation occurs on lysine residues of the targeted substrates. There are 64 lysines in DENV NS5 protein that might serve as the SUMO acceptors. To delineate the SUMOylated residues in NS5, various HA-tagged NS5 truncation mutants were generated (Fig. 3A) and subjected to *in vivo* SUMOylation assay. Given that the SUMO modification machinery is predominantly nuclear and that NLS are important for the SUMOylation of nuclear proteins (19, 41), we preserved the NLS in all of these deletion mutants. By *in vivo* SUMOylation assay, we found that EGFP-tagged SUMO1 was only detected on the NS5 fragments containing the 71 to 300 residues at the N terminus (Fig. 3B, lanes 1 to 8), whereas the C-terminal half of NS5 seemed not to be critical for SUMOylation (Fig. 3B, lanes 9 to 14). These results suggested that two acceptor lysine sites for SUMOylation are located in the N-terminal 71 to 300 residues of NS5.

To finely map the SUMOylation sites, we examined each lysine residue within the N terminus. The lysine residues within the 71 to 300 amino acids in the predicted SUMO target region of DENV NS5 protein are shown in the schematic diagram in Fig. 3C. Although there are 14 lysines in this region, the inspection of the amino acid sequence revealed that none of the lysine residues contains the postulated SUMOylation motif (42). Nevertheless, we performed site-specific mutation on all of the 14 lysines (K to R) in the full-length NS5 protein individually or in groups. However, none of the mutants lost the ability to be SUMOylated (Fig. 3D and E). Even when all 12 lysines residues between 100 and 300 amino acids were mutated, namely, the 12(K-R) mutant (Fig. 3G, lanes 3 and 4), there was still a weak SUMOylated band. These results suggested that SUMOylation of NS5 floats within this N-terminal region, namely, the loss of one SUMOylation site likely is compensated for by SUMOylation on other residues. We did not characterize further the nature of SUMOylation on these mutants. Instead, we attempted to identify the SUMO-interacting motif (SIM), which has been reported to consist of a hydrophobic core with the consensus sequence V/I-X-V/I-V/I (43), that mediates noncovalent interaction with SUMO and regulates protein SUMOylation (19, 21, 44, 45). By protein sequence alignment of four different DENV serotypes, a potential SIM harboring conserved V-V/I-D-L (amino acids 77 to 80) was found in the predicted SUMO target region of NS5 (Fig. 3F). To determine the effect of SIM on NS5 protein SUMOylation, a SIM mutant containing the VVDL-to-AAAA substitution was generated and subjected to *in vivo* SUMOylation assay. In contrast to the WT, the SUMOylation was completely abolished in SIM-mutated NS5 (Fig. 3G, lanes 5 and 6), suggesting that this N-terminal SIM motif is required for NS5 protein SUMOylation.

### NS5 SUMOylation regulates the replication of DENV replicon.

**FIG 4:**
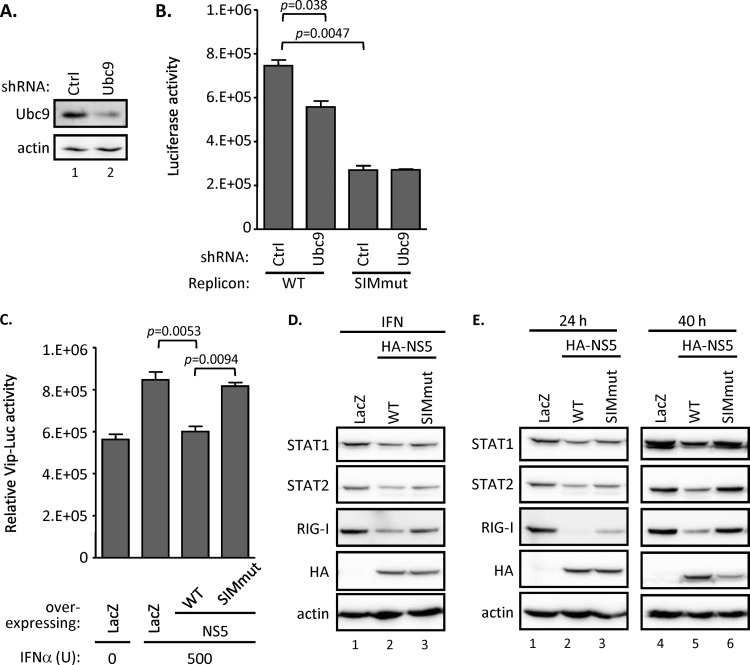
SUMOylation of DENV NS5 is required for the replication of DENV replicon and for the antagonism of IFN signaling. (A) Endogenous Ubc9 protein expression level in Huh7 cells stably expressing shRNA targeting control (Ctrl) or *Ubc9* (Ubc9) were analyzed by WB. (B) Huh7 cells stably expressing shRNA targeting Ctrl or *Ubc9* were transfected with DENV replicon RNA harboring WT or SIM-mutated NS5 as indicated. The cell lysates were harvested and analyzed by luciferase assay after 24 h of transfection. The error bars represent the means and SD (*n* = 3 per group) and were compared by Student's *t* test. (C) A549 cells were cotransfected with Vip-Luc (0.3 μg) and pRL-TK (0.1 μg) plus LacZ control, WT, or SIM-mutated DENV NS5 (0.5 μg) for 16 h. Subsequently, transfected cells were treated with IFN (500 U) or left untreated (0 U) for 8 h. The cells were harvested and analyzed by dual-luciferase assay. Data are expressed as means and SD (*n* = 3 per group) and were compared by two-tailed Student's *t* test. (D) A549 cells were transfected with LacZ control, WT, or SIM-mutated NS5 expression plasmids for 16 h, followed by another 8-h treatment of IFN (500 U). The cells were collected and analyzed by WB with the indicated antibodies. (E) A549 cells were transfected with LacZ control, WT, or SIM-mutated NS5 expression plasmids for 24 or 40 h and then analyzed by WB using the indicated antibodies.

Since the SIM motif is required for NS5 SUMOylation, we next determined its potential biological roles in DENV infection. Huh7 cells stably expressing the control shRNA (Ctrl) or Ubc9-targeting shRNA (Fig. 4A) were transfected with *in vitro*-transcribed RNA derived from WT or a SIM-mutated DENV subgenomic replicon containing a luciferase reporter gene, which reflects the viral RNA replication level. By measuring the luciferase activity, we found that the replication level of WT replicon was consistently and significantly lower in the cells stably expressing Ubc9-targeting shRNA than that of control shRNA (Fig. 4B), suggesting that Ubc9 activity is required for DENV RNA replication. In contrast, with the SIM-mutated replicon, the luciferase activity was further reduced in both the control and Ubc9 knockdown cells. Collectively, these results suggested that Ubc9-mediated SUMOylation is required for DENV RNA replication, and this posttranslational modification relies on the presence of the putative SIM motif of DENV NS5.

In addition to its role as an RNA polymerase in DENV RNA replication, NS5 has been shown to mediate the virus-induced suppression of IFN signaling (5, 6, 46, 47), thereby enhancing virus replication. To determine whether the DENV NS5-mediated suppression of IFN signaling requires SUMOylation, we used Vip-Luc (37), a luciferase reporter whose expression is driven by the promoter of the interferon-stimulated gene viperin, to evaluate the potency of IFN responses. Consistent with previous studies, we found that IFN-induced Vip-Luc activity was suppressed in the cells transfected with WT NS5 relative to that in the LacZ-transfected cells. In comparison, SIM-mutated NS5 failed to inhibit the Luc activity (Fig. 4C) and failed to suppress the expression of the IFN-inducible gene RIG-I (Fig. 4D). Since the interferon-inhibitory effect of DENV NS5 previously has been linked to STAT2 degradation (5, 6, 46, 47), we then examined the protein expression level of endogenous STAT2 and found that the STAT2 level was higher in cells expressing the SIM-mutated NS5 than those expressing WT NS5 (Fig. 4E, lanes 2, 3, 5, and 6). Interestingly, the amounts of STAT1 and RIG-I also were higher in the cells expressing the mutant NS5 (Fig. 4E). These results support the conclusion that a functional SIM sequence is important for the NS5-mediated suppression of IFN signaling and action.

### The SUMOylation stabilizes NS5.

**FIG 5:**
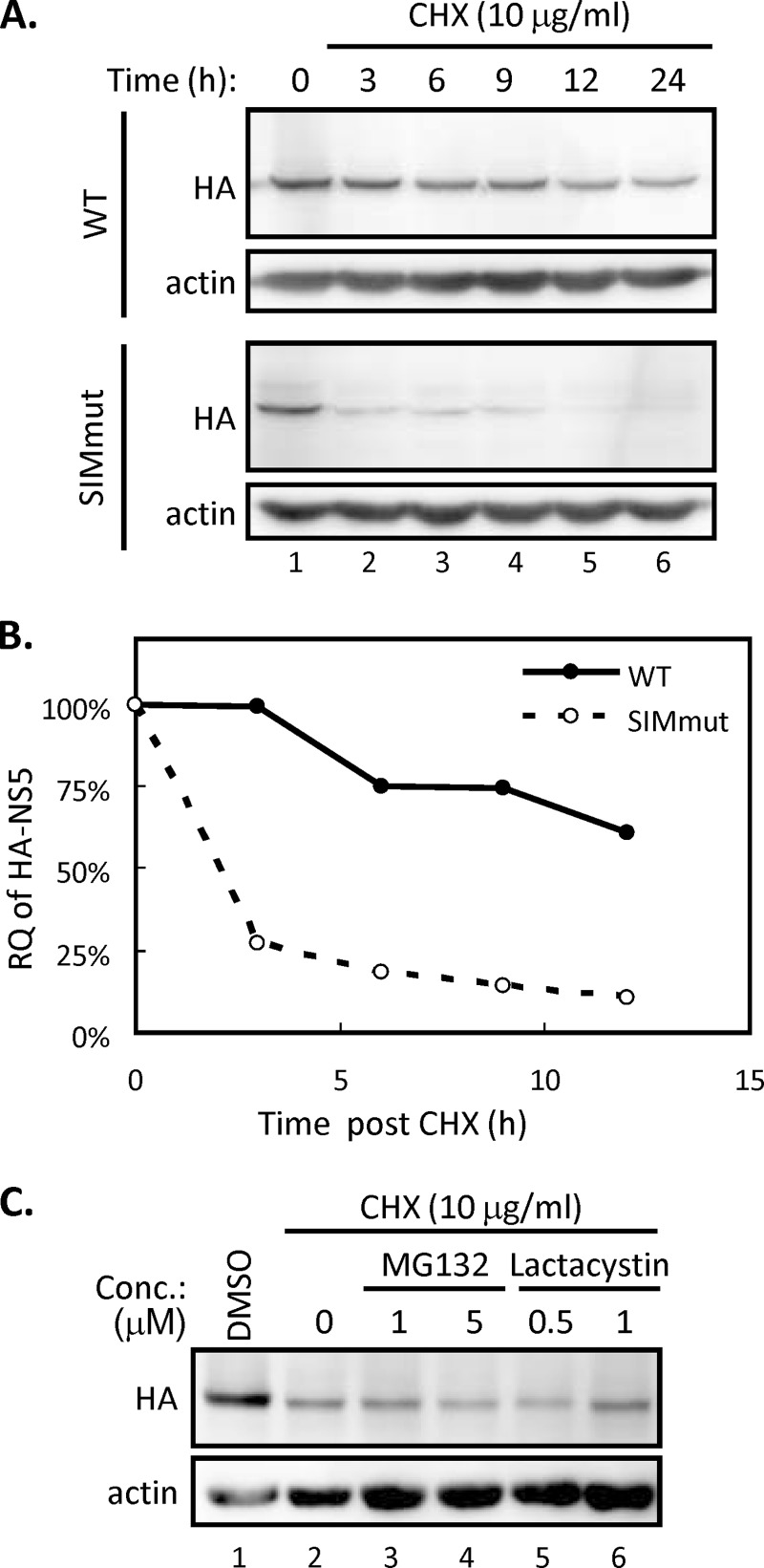
SUMOylation regulates the stability of NS5. (A and B) BHK-21 cells were transfected with WT or SIM-mutated NS5 for 24 h and then treated with cycloheximide (CHX). Cells were harvested at different time points after CHX treatment as indicated. (A) The NS5 protein expression levels were analyzed by WB using antibodies against HA and actin. (B) The band intensity of NS5 was quantified by ImageJ and normalized to that of actin. The relative quantities (RQ) were calculated and are shown as percentages of the quantities at 0 h. (C) BHK-21 cells were transfected with SIM-mutated NS5 for 24 h, designated hour 0, and then either left untreated or treated with CHX (lanes 2 to 6) together with the indicated inhibitors for another 3 h. Cells were harvested and analyzed by WB with anti-HA and anti-actin antibodies.

It is noted that the protein expression level of SIM-mutated NS5 was almost the same as that of the WT at 24 h posttransfection but significantly lower than that of the WT at 40 h posttransfection (Fig. 4E, lanes 2, 3, 5, and 6). This finding raised the possibility that SUMOylation stabilizes DENV NS5, thereby enhancing the biological activity of NS5. To check this possibility, A549 cells transfected with WT or SIM-mutated NS5 were treated with cycloheximide (CHX), an inhibitor of protein translation. We found that the amount of WT NS5 protein remained at a high level throughout the 24-h period after CHX treatment, whereas the SIM-mutated NS5 protein degraded rapidly in the same period (Fig. 5A). The quantitative analysis of the kinetics of protein reduction showed that WT NS5 was more stable than the SIM mutant in A549 cells (Fig. 5B). We found that protein expression levels of HA-tagged, SIM-mutated NS5 after CHX treatment was not recovered by either reversible MG132 or irreversible lactacystin, the chemical inhibitors targeting proteasomal machineries (Fig. 5C). Thus, our data suggested that the failure of NS5 to be SUMOylated lowers the stability of NS5 and leads the latter to degradation through proteasome-independent machinery.

Taken together, these results showed that DENV NS5 requires a functional SIM motif to stabilize itself for its biological functions, and that NS5 SUMOylation may regulate DENV replication by governing the protein turnover of the replication complex.

## DISCUSSION

SUMOylation has emerged as a central regulator controlling protein function under physiological or stressful conditions (48, 49). In the last decade, increasing data have concerned the interaction between various viruses and SUMO machinery (22, 28). In this study, we provided evidence showing that DENV NS5 is a SUMOylated protein, and the fate and function of the protein were governed by SUMOylation, which is critical for DENV replication. The SUMOylation of NS5 protein may be transient and can be removed by SENP1 and SENP2. Thus, it is highly dynamic, so that only a portion of NS5 is SUMOylated in DENV-infected cells. We further showed that failure to modify NS5 with SUMO destabilizes NS5; as a result, NS5 could not support DENV replication and also lost its ability to suppress innate immunity. Thus, NS5 SUMOylation represents an important mechanism for proper viral functions. Whether other cellular or viral factors orchestrate NS5 function by SUMO-mediated interaction remains to be elucidated.

Several previous studies have suggested the involvement of the cellular SUMOylation pathway in DENV replication. By a computational approach implementing the protein interaction networks between DENV gene products and host factors (38), the cellular SUMO E2 ligase Ubc9 has been predicted to interact with several DENV proteins. By a yeast two-hybrid study, Ubc9 also was found to interact with several DENV nonstructural proteins, such as NS2B, NS4B, and NS5 (34). However, we found that only NS5 could be SUMOylated. We also showed that the amounts of viral protein in DENV-infected cells were decreased by silencing Ubc9; correspondingly, the replication levels of DENV replicon were reduced by introducing the SUMOylation-defective NS5. These data together support the scenario that the SUMOylation system is involved in the DENV life cycle. Given that the components of the SUMOylation pathway have been identified in Drosophila melanogaster (50) and DENV replicates and is transmitted by mosquitos, whether the SUMOylation system participates in the susceptibility to DENV infection in certain mosquitos (51) will be of great interest to public health.

Among the 10 DENV proteins we tested, only NS5 protein can be conjugated with SUMO. Despite the fact that DENV RNA replication occurs in the cytoplasm, the subcellular localization of DENV replicase NS5 is predominantly in the nucleus (19, 41, 52), where the SUMOylation machineries are mainly located. We found that the SIM mutant of NS5 can be readily detected in the nucleus and an NLS-mutated, cytoplasm-located NS5 can be SUMOylated (not shown), indicating that SUMO is not the factor responsible for NS5 nuclear localization. We have demonstrated that SUMOylation occurs within the N terminus of NS5 but failed to identify the individual lysine residues responsible for this SUMOylation despite the large number of lysine mutants generated in our laboratory. This failure suggests the presence of alternative and floating SUMOylation sites within NS5. This was also the case for the SUMOylation of the cellular transcriptional repressor DAXX (44) and HDV nuclear protein hepatitis delta antigen (HDAg) (30). NS5 of yellow fever virus (YFV) also has been reported to be ubiquitinated after IFN treatment. In that case, any lysine residues in the first 10 residues of the N-terminal region in YFV NS5 could be the alternative ubiquitin acceptor site for this IFN-triggered K63-linked polyubiquitination (53). We propose that the SUMO acceptor site of DENV NS5 will be relocated once the primary lysine target site is artificially mutated. Thus, the site of SUMOylation could be floating within certain protein domain(s).

For a virus-like DENV, which employs the viral protein expression strategy from a single open reading frame, SUMOylation of NS5 may be one of the strategies to fine-tune protein turnover to remove the excess viral replicase in host cells. In this regard, we have identified a SIM motif that is responsible for DENV NS5 SUMOylation. The SIM motif initially was identified as an S-X-S motif surrounded by hydrophobic and acidic amino acids (54). However, later studies demonstrated that the core sequence of SIM should encode one acidic residue and a stretch of three or four hydrophobic residues characterized as H-X-H-H or H-H-X-H (43, 55). SIM has been shown to be necessary for promyelocytic leukemia-nuclear body formation (56) and for the SUMOylation and transcription of DAXX (44). These data indicated that SIM modulates the cellular functions of SUMO-binding proteins. By using the SUMOylation-defective NS5 mutant of DENV replicon, we showed that the SUMOylation of NS5 protein is required to regulate the replication of DENV replicon by increasing the stability of the protein.

The reported functions of protein SUMOylation are extremely diverse, ranging from alteration in intracellular localization to the changing activity of the modified proteins (15, 16, 57). DENV NS5 protein is an RNA replicase containing two functional domains: the N-terminal MTase domain binds GTP and performs methylation reactions required for viral RNA cap formation (9), whereas the C-terminal RdRp domain is responsible for the synthesis of the DENV RNA genome (4). We found that the SUMO target region and SIM were located at the N-terminal MTase domain. We do not know whether the SUMOylation of NS5 altered its MTase activity or RdRp activities directly. Nevertheless, we showed that the SIM mutants are unstable due to their short half-lives. The main function of SUMOylation may be to stabilize the SUMOylated NS5, thereby indirectly accounting for most of its reported biological properties. The *de novo* DNA methyltransferase 3a (Dnmt3a), one of the DNA MTases in mammal cells, also was identified as a SUMO target protein. SUMOylation of Dnmt3a disrupts its ability to interact with histone deacetylases and abolishes its capacity to repress transcription (58). Another DNA MTase, DNMT1, also was found to interact with Ubc9, to be modified by SUMO, and to enhance its methylase activity by SUMOylation (59). Whether NS5-SUMOylation has similar direct effects on the enzymatic activities remains to be seen. For comparison, the SUMOylation of NS5A of hepatitis C virus may be directly involved in RNA replication (60). Interestingly, in the case of HCV, the SUMO-defective mutant (K348R) was continuously reverted to the wild-type sequence during viral replication, providing additional evidence that SUMOylation is required for HCV replication. If SUMOylation is essential for DENV survival, it is to the advantage of the virus to have multiple lysine residues serving as SUMO acceptors within NS5 protein.

Another function of DENV NS5 is inhibiting IFN functions by degrading STAT2, leading to the subsequent inhibition of innate immunity (5, 6). The loss of this ability by the SUMOylation-defective mutant most likely is the result of NS5 protein degradation. SUMO modification of HCV NS5A can increase the protein stability by inhibiting ubiquitination and contributes to HCV replication (60). We found that the DENV NS5 harboring a defective SIM was unstable, failed to degrade STAT2, and was unable to support DENV replication. Therefore, SUMO modification may govern DENV NS5 stability, regulating DENV replication and affecting host antiviral signaling. On the other hand, the differential phosphorylation of DENV NS5 would modulate its interaction with NS3 and determine the subcellular localization of NS5 in infected cells (13). Whether the function and/or activity of DENV NS5 can be regulated posttranslationally by phosphorylation and SUMOylation of various viral proteins in a sequential or synergistic manner requires further study.
